# Large Chest and Abdominal Wall Defect Reconstruction With Anterolateral Thigh Free Flap to Right Gastroepiploic Artery Anastomosis

**Published:** 2017-09-18

**Authors:** Angie Zhang, Amra Kuc, Paul Smith, Amanda Zimmerman, Alicia Billington, Ricardo J. Gonzalez, Deniz Dayicioglu

**Affiliations:** ^a^Morsani College of Medicine, University of South Florida, Tampa; ^b^Division of Plastic Surgery, Department of Surgery, Morsani College of Medicine, University of South Florida, Tampa; ^c^Sarcoma Department, H. Lee Moffitt Cancer Center and Research Institute, Tampa, FL, USA

**Keywords:** anterolateral thigh free (ALT) flap, recurrent metaplastic sarcomatoid carcinoma (MSC), gastroepiploic artery anastomosis, chest and abdominal wall reconstruction, tumor resection defect

## DESCRIPTION

A 39-year-old woman presented with a twice-recurrent, full-thickness left chest and abdominal wall metaplastic sarcomatoid carcinoma (MSC). Two additional radical resections of the tumor left a defect measuring 30 × 40 cm. An anterolateral thigh (ALT) free flap with anastomosis to the right gastroepiploic vessels was chosen for reconstruction.

## QUESTIONS

1. What are the local reconstructive options for chest and abdominal wall reconstruction?
2. What are the free flap options?
3. What considerations were made in the reconstruction of a patient with a large defect and vital organ exposure?
4. Which vessels are used as a recipient in free tissue transfer in abdominal reconstruction?

## DISCUSSION

Advances in reconstructive surgery over the past 2 decades have permitted the oncologist to proceed with radical cancer resection and ensure negative margins without the fear of causing an irreparable defect. Local options in defect reconstruction include primary closure, skin grafting, tissue expansion, negative pressure-assisted closure, the components separation technique, prosthetic mesh, and pedicled flaps.^1^^,^^2^ Primary closure and skin grafting are methods suitable for smaller, partial-thickness defects. For full-thickness coverage, serial tissue expansions can provide reliable coverage with excellent aesthetic outcomes.^2^^,^^3^ Negative pressure–assisted closure can be used for temporary coverage in delayed reconstruction as well as stimulation of healing by secondary intention.^4^ It also allows delay of the ultimate reconstruction by ensuring negative margins in a sarcoma excision. Component separation with or without mesh provides tissue strength, but regional or local flaps would also need to be utilized for the superficial fascial layer reconstruction.^5^ Prosthetic mesh or human acellular matrix utilization is important in attaining chest wall stability.^6^

Free flaps are indicated when there is a massive loss of local tissues or when the defect size exceeds the reach or coverage of a pedicled flap.^7^ Free flaps include the latissimus dorsi, the omentum, the tensor fascia lata, the rectus femoris muscle flap, and the ALT flap. Latissimus dorsi flap limitations include limited upper extremity motion, donor site morbidity, and the need for skin grafts. The use of omentum provides the benefits of visceral protection and a reliable blood supply, but it often requires skin grafting and repair of a potential hernia. A tensor fascia lata flap is the preferred flap in the reconstruction of defects involving the lower two-thirds of the abdominal wall due to the strength it provides to that area. A free ATL flap with gastroepiploic artery anastomosis is capable of reliably reconstructing complex, full-thickness chest and abdomen defects in a single stage without the use of vein grafts. In addition, a free ALT flap has low donor site morbidity and allows the surgeon to harvest it with minimal amounts of skin, fascia, and muscle necessary.^2^

**Figure 1:**
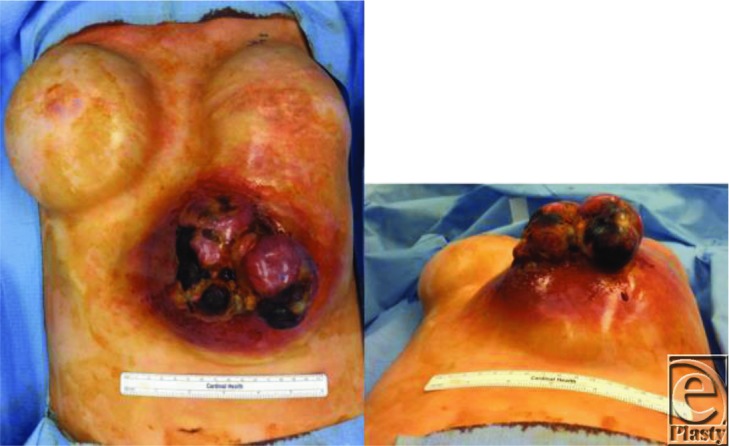
Preresection views of the necrotic, fungating, twice-recurrent, metaplastic sarcomatoid carcinoma. The patient was diagnosed at the age of 29 years at which time she underwent resection and bilateral mastectomies with implant placements. At the age of 35 years, she had a recurrence and another resection. At the age of 37 years, a second recurrence was noted. Tumor was not resected, and the patient refused radiation therapy at that time. Two years later, the patient sought medical therapy from our team.

**Figure 2:**
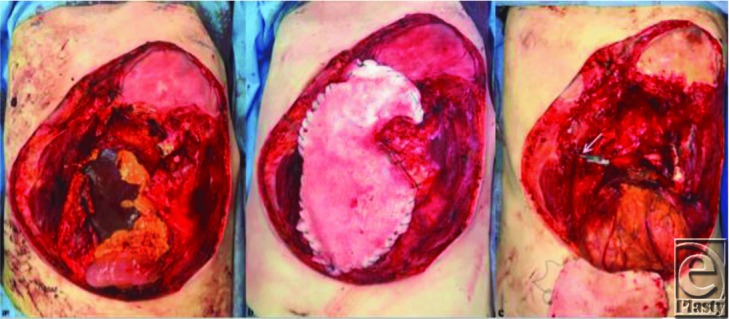
(a) Postresection, including removal of breast implants, prior to Strattice (porcine-derived surgical mesh) placement. (b) Postresection defect with Strattice in place. Wound VAC was applied postoperatively. Delayed plastic surgery defect reconstruction to take place following confirmation of negative margins. (c) Intraopeartive view of the defect after negative margins were obtained. The arrow identifies the exposed right gastroepiploic artery.

**Figure 3:**
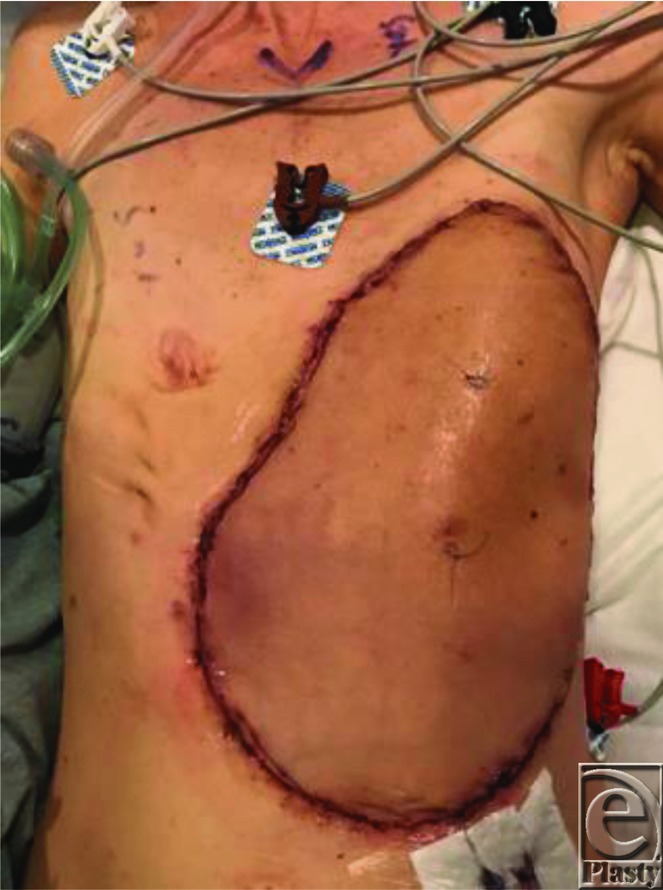
Immediately postoperatively.

**Figure 4:**
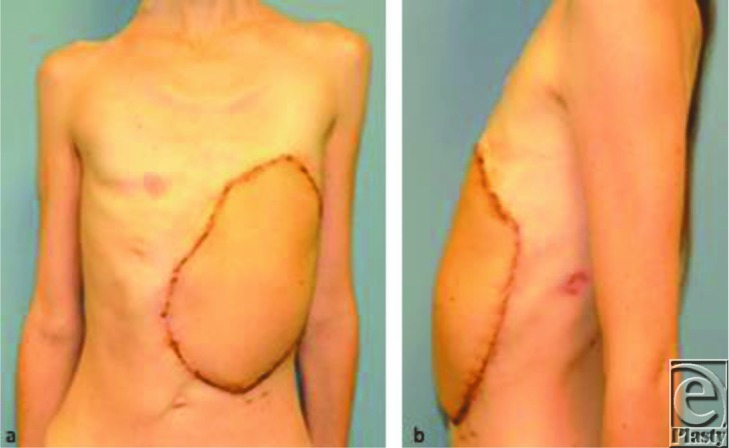
Three months postoperatively. (a) Anterior view. (b) Lateral view.

Our patient was a 39-year-old woman who presented with a twice-recurrent, full-thickness, left chest and upper abdominal wall MSC involving the costal margin and costochondral junctions of multiple lower ribs (Fig 1). The patient underwent radical resection of the 18 × 20-cm ulcerated tumor with en bloc removal of skin, subcutaneous tissues, costal margin, and left rectus margin. Upon positive margins, she further underwent reexcision of the deep superior and deep lateral margins, leaving a defect measuring 30 × 40 cm (Figs 2*a* and *b*). In planning reconstruction, the extensive musculofascial damage and defect dimensions precluded our use of the rectus abdominis and the latissimus dorsi, respectively, so we chose to proceed with a free flap consisting of the contralateral ALT and a part of the vastus lateralis muscle. Incorporation of the vastus lateralis allowed for a larger skin paddle and its added bulk is beneficial for dead space obliteration.^7^ Since the internal mammary artery was sacrificed during dissection along the lower ribs, the right gastroepiploic vessels were identified as the most optimal recipient vessels as they had already been exposed during dissection (Fig 2*c*). The descending branch of the circumflex femoral artery was anastomosed to the right gastroepiploic artery through an opening on the posterior rectus sheath, over the liver, so as to avoid any undue tension or hernia formation (Figs 3 and 4).

In abdominal reconstruction, recipient vessels typically utilized are the extraperitoneal vessels, including the deep epigastric vessels, the internal mammary vessels, and the saphenous vein loop graft; however, intraperitoneal vessels, such as the gastroepiploic vessels, may also be used.^7^^,^^8^ The intraperitoneal vessels, due to their location immediately deep to the defect, allow for the use of free flaps with short pedicles or wide and flat flaps. In addition, they allow for a tight, continuous, circumferential fascial closure that is not topologically achievable with extraperitoneal vessels. The intraperitoneal gastroepiploic vessels, in particular, have high flow rates, diameters of 2 to 3 mm, and are easily identified and dissected.^8^

Successful reconstruction is contingent on fully addressing the anatomical, functional, and cosmetic deformities of a defect. Our case demonstrated reconstruction of a complex wound in a single stage using the gastroepiploic artery for anastomosis.
